# Motion compensated magnetic resonance imaging of an active sun beetle using an in situ treadmill

**DOI:** 10.1038/s41598-025-27800-5

**Published:** 2025-11-18

**Authors:** Ajmal Chenakkara, Mazin Jouda, Ulrike Wallrabe, Jan G. Korvink

**Affiliations:** 1 Institute of Microstructure Technology (IMT), Karlsruhe Institute of Technology, Karlsruhe, Germany; 2 Institute of Microsystem Technology (IMTEK), University of Freiburg, Freiburg, Germany

**Keywords:** Mechanical engineering, Biomechanics

## Abstract

Magnetic resonance imaging is inherently non-invasive, and thus an ideal technique for probing living biological matter. The low sensitivity and prolonged data acquisition time, coupled with stringent magnetic field homogeneity requirements and spatial constraints inside the magnet, make the technique under-utilised for the study of live and freely moving model organisms. We introduce a new method for performing MRI of a live insect that is moving on a treadmill. The tethered insect, positioned on a treadmill inside an RF volume coil, provides a controlled environment for studying the organism, and maintains the spatial consistency for an MRI excitation slice, thereby limiting any residual motion artifacts within the slice or MRI field-of-view, and thus making the problem manageable with motion correction techniques available in clinical MRI research. We address the particular case of semi-periodic abdominal motion of the insect, and its effect on MRI reconstruction. An MR compatible optical imaging system has been integrated with the high-field magnet, in conjunction with a computer vision algorithm for extracting the real-time motion information, with the added advantage of phenotypic characterisation of the behaving organism. The motion information, with a prospective triggering system, has been used for the acquisition of spatially consistent k-space lines, thereby reducing artifacts due to the gross body motion of the walking insect.

## Introduction

Magnetic Resonance Imaging is a non-invasive tool that provides rich morphological, functional, chemical, and dynamic information of living biological matter. The sensitivity of MR-based techniques is inherently low and primarily relies on sequential acquisition with extensive signal averaging. Three orthogonal gradient coils define the spatial coordinate system for MRI. Motion of the test subject causes a model mismatch between the subject’s spatial coordinates and the MRI coordinate system during the sequential acquisition of MR signals (k-space lines) and causes artifacts upon image reconstruction, hence the problem of motion in MRI. This presents a fundamental limitation for studying freely behaving model organisms using MRI. Present-day techniques involve sacrificing^1^, physical restraining^2^, or anesthetizing the organism, which alters the natural behavior of the specimen. Prospective motion correction strategies where the MRI field of view is readjusted to the ongoing motion of the subject are quite successful techniques in the clinical MRI^3,4^ research for rigid body motion correction. This strategy is quite limiting and challenging in the case of the natural behavior of the small organisms due to the spatial constraints of the RF and gradient field coils, in addition to the problem of maintaining spatial $B_0$ and $B_1$ field homogeneity. The possibility of such an approach has been explored in silico for the particular case of *C. elegans* as the model organism^5^.

The impact of motion in MRI strongly depends on the specific technique and sequence used. In MR microscopy, the issue is more severe due to lower sensitivity from small sample volumes, often requiring signal averaging over several hours. The problem of motion and its effects and correction techniques in the clinical MRI domain has a rich literature and reviewed elsewhere^6,7^. Here, we present a novel approach for applying MR-based techniques to a live, freely walking insect by integrating an in situ spherical treadmill within a high-field MRI system (15.2 T Bruker BioSpec; Bruker BioSpin GmbH, Ettlingen, Germany). The tethered insect walks on an air-suspended ball, with the entire setup integrated within an RF volume coil. This provides a controlled environment for studying the internal anatomy of the live freely walking insect using MRI. A full-body restraint can suppress natural behavior including locomotion, respiration, and muscle dynamics, thereby limiting physiological relevance. In contrast, our method minimizes restraint to what’s necessary for MRI stabilization while permitting active locomotion, enabling more naturalistic in situ experiments. Treadmill-based experimental paradigms are well established across various biological disciplines, including biomechanics, exercise physiology, metabolic studies, and neuroethology^8–11^. A common objective across these fields is to create a relatively stress-free environment that allows for free movement and spatial consistency, thereby facilitating reliable access to experimental instrumentation and protocols for studying the organism. In parallel, there has been significant progress in awake small-animal fMRI research aimed at enabling functional brain imaging without the confounding effects of anesthesia^12–14^. However, many of these studies still rely on full physical restraints and pre-scan anesthesia to minimize motion during imaging. Additionally, advances in flow-through MRI systems have demonstrated the feasibility of non-invasive, high-resolution imaging of physiological responses in live fish and marine invertebrates exposed to environmental challenges under controlled, simulated conditions^15–17^. However, to the best of our knowledge, there appears to be limited progress in developing integrated platforms for MRI of behaving animals, especially at the scale of insect sizes presented in this study. Advancements in the field of MRI method development has been heavily concentrated on human clinical applications and research involving other vertebrates like rodents and non-human primates. Invertebrate model organisms are advantageous for research due to their rapid life cycles, cost-effectiveness, and powerful genetic tools, which allow for efficient study of fundamental biological processes.

While the treadmill setup offers a controlled environment for studying active organisms, they inevitably introduce motion artifacts, primarily due to gross abdominal movements. Accurate estimation and characterization of such motion is critical for effective MRI motion correction. A wide array of motion estimation strategies has been developed in clinical MRI research, encompassing both internal MR-based methods^18,19^ and external hardware-based techniques^6^. Among these, optical imaging systems have emerged as powerful tools for extracting real-time motion data in an MRI-sequence-agnostic manner, as demonstrated in several studies^3,20,21^. These systems include multi-camera setups positioned outside the magnet bore^3,22^, MR-compatible cameras within the bore^23^, and even camera systems integrated into the RF coil^20^. Notably, it has also been shown that general-purpose optical microscopy can be directly integrated with high-field MR scanners, enabling simultaneous MR and optical imaging^24,25^. Providing an optical imaging access to the treadmill setup facilitates real-time motion estimation and monitoring of the organism’s behaviour. In this study, we developed an optical imaging system with a computer vision algorithm optimized for use within the high-field magnet environment. Traditional optical tracking methods typically rely on rigidly attached markers to the subject’s body^3,26,27^, which is both invasive and challenging to implement for freely moving insects. To overcome this, we implemented a sparse optical flow algorithm that accurately tracks user-defined insect body parts without markers, enabling precise motion field estimation across video frames. We further characterized the system’s detection performance, tracking accuracy, and latency. Additionally, we developed a prospective gating strategy that leverages 2D motion data from the computer vision system to selectively acquire k-space lines during motion-consistent periods, effectively minimizing artifacts from the insect’s movements. Such gating techniques, commonly used in clinical and preclinical MRI, synchronize data acquisition with physiological signals like respiration or cardiac cycles to improve image quality by reducing motion-related distortions^28–30^.

In essence, our methodological contribution lies in adapting a motion compensation strategy to treadmill-based experiments within a high-field MRI system. As a proof-of-concept, we selected *Pachnoda marginata*, a species that is readily available, large enough to yield a strong and reliable MRI signal, and slow-moving enough to facilitate the development and validation of our motion compensation technique. Our approach establishes a foundation for future MR-based investigations involving widely studied yet challenging model organisms such as *Caenorhabditis elegans* or *Drosophila melanogaster*.

**Figure 1:**
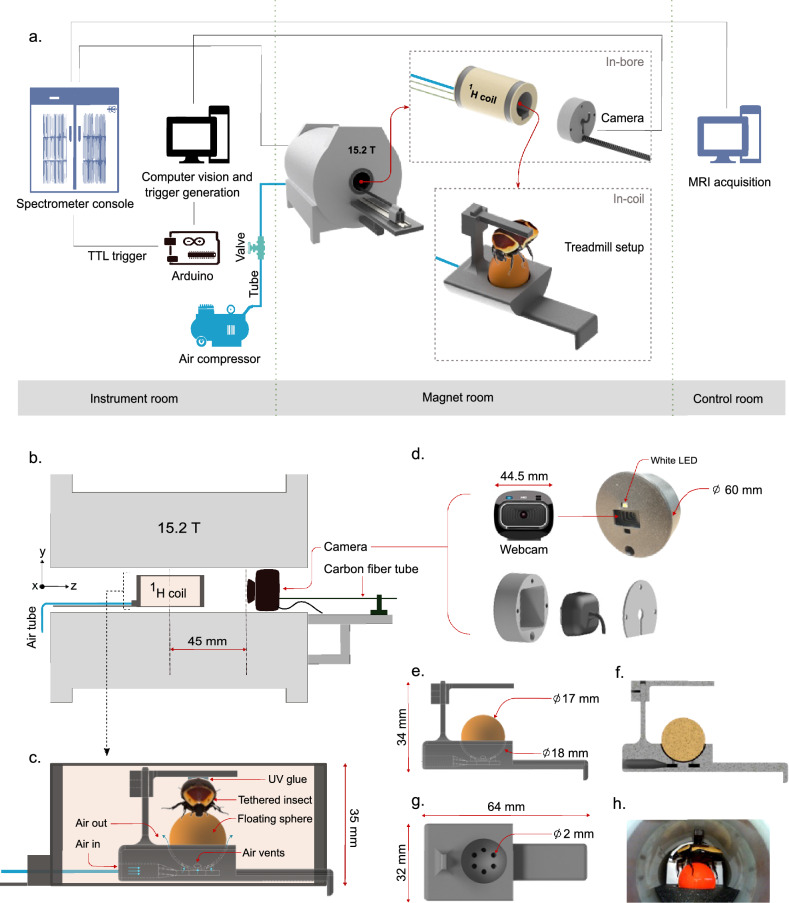
Design and development of treadmill setup. (**a**) Schematic diagram of in situ treadmill with integrated computer vision system for prospective triggering. (**b**) Integrated treadmill with optical imaging system inside 15.2 T magnet. (**c**) Spherical treadmill setup inside the $^{1}{H}$ RF coil (figure created using Inkscape v1.3.2 (https://inkscape.org)). (**d**) MR compatible optical imaging system. (**e–g**) CAD rendering of the treadmill assembly. (**h**) Treadmill with live beetle in situ $^{1}H$ RF coil.

## Materials and methods

### In situ treadmill experimental setup

The treadmill setup consists of a 3D printed (Prusa MK4 printer with Prusament PLA filament, Prusa Research a.s., Prague, Czech Republic) structure with overall assembly dimension of ${64}\hbox { mm} \times {32}\hbox { mm} \times {34}\hbox { mm}$, which holds a sphere of diameter 17 mm, as shown in the Fig. 1a, c, e. The structure consists of a hemispherical slot of 18 mm diameter with 6 air vents of 2 mm diameter for suspending the ball within a cushion of air, shown in Fig. 1e–g. The insect is tethered on a vertically adjustable support structure using a UV-cured glue (Delo Industrial Adhesives GmbH & Co. KG, Windach, Germany), and is suspended on top of the sphere. The air-cushioned ball provides a frictionless platform, enabling the insect the ability to walk freely in any direction relative to its fixed body. Dry air supply is tapped out from a laboratory supply using PVC tubing of 4 mm inner diameter, and a pneumatic shut-off valve.

The whole setup is integrated inside a 35 mm inner diameter quadrature $^{1}H$ RF volume coil of bird cage configuration, which is tuned and matched to 650 MHz for the 15.2 T magnet, as shown in Fig. 1b, c. Figure 1e–g shows the CAD render (Autodesk Inventor v2023, Autodesk Inc., CA, USA) of the treadmill setup. Figure 1h shows the in situ behaving insect on the treadmill setup. The configuration of the treadmill is modular, allowing easy placement inside the volume coil to position the insect at the coil’s isocenter. The PLA-based 3D printed structure is fully compatible with high magnetic fields and did not show any detectable susceptibility artifacts during the MRI acquisition. The treadmill provides an infinite plane for the insect to move around while maintaining a fixed in-plane orientation for the FOV, and $B_0$ and $B_1$ field homogeneity for the MR experiments.

### MR-compatible optical imaging system

The real-time motion information and physiological response of the insect inside the high-field magnet can be obtained using an integrated optical imaging setup. The optical imaging and illumination system has been developed using a commercial webcam (Microsoft LifeCam HD-3000, Microsoft Corporation, Redmond, WA, USA) with a maximum frame rate of 30 Hz, image resolution of $1280 \times 720$ pixels and a white LED, which is mounted as a single unit using 3D printed (Prusa MK4 printer with Prusament PLA filament, Prusa Research a.s., Prague, Czech Republic) parts as shown in Fig. 1d. The camera and LED are powered from the USB connection with the computer (Lenovo Thinkpad E14, Lenovo Group Limited, Beijing, China), which is used for grabbing the live video feed and image processing for the gating trigger signal. A USB active repeater cable of length 20 m is used for connecting the in situ camera to the computer in the instrument console room. The manual focus ring has been utilized to precisely adjust the camera’s focus and placed the camera system in horizontal alignment with the RF coil at a distance of 45 mm from the center of the RF coil inside the magnet bore, as represented in the Fig. 1b. A carbon fiber tube of length 1 m has been attached to the imaging setup for accessibility inside the magnet bore. The system is made MR-compatible by removing the magnetic parts from the webcam (further details on the development of the imaging system are provided in the “MR-compatible camera” section of the Supplementary Information). The effect of placement of the camera inside the magnet on the MRI experiments has been studied using an MRI phantom sample setup, details of which are provided in the “Effect of in situ optical imaging system on MRI” section.

We observed no RF interference or data corruption from the camera during MRI acquisition. The camera uses digital USB transmission with differential signaling and shielding, which reduces susceptibility to electromagnetic interference. The RF excitation pulse had an estimated amplitude of $B_{1}(t) \approx {3.74}{\upmu \hbox { T}}$, based on a sinc-modulated waveform with a duration of 1.575 ms, a bandwidth of 2 kHz, and a flip angle of ${90}^{\circ }$. We infer that the combination of camera placement outside the coil, low RF power, digital transmission, and shielding was sufficient to prevent interference; however, more intense RF or gradient pulses, or the use of unshielded cables, may produce different outcomes. Video file showing in situ tracking can be found in the supplementary information (supplementary_video.mp4).

### Optical flow based tracking

We have used a sparse optical flow based algorithm for tracking predefined single point (posterior part of the insect abdomen) motion. Optical flow is defined as the distribution of apparent velocities of movement of brightness patterns in an image. The basic assumption is that the intensity of a point in the image remains constant over time, leading to the brightness constancy constraint. This can be mathematically expressed as:

1 $$\begin{aligned} I(x, y, t) = I(x + \delta x, y + \delta y, t + \delta t). \end{aligned}$$

Here $I(x, y, t)$ is the image pixel intensity at position $(x, y)$ and time $t$. Expanding this using a Taylor series and ignoring higher-order terms gives:

2 $$\begin{aligned} I(x + \delta x, y + \delta y, t + \delta t) \approx I(x, y, t) + \frac{\partial I}{\partial x} \delta x + \frac{\partial I}{\partial y} \delta y + \frac{\partial I}{\partial t} \delta t. \end{aligned}$$

Since the intensity is assumed to be constant:

3 $$\begin{aligned} \frac{\partial I}{\partial x} u + \frac{\partial I}{\partial y} v + \frac{\partial I}{\partial t} = 0. \end{aligned}$$

Here, $u = {\delta x}/{\delta t}$ and $v = {\delta y}/{\delta t}$ are the components of the optical flow vector, equation (3) is known as the optical flow constraint equation (OFCE). One common method to solve the OFCE is the Lucas-Kanade method^31^. It assumes that the flow is essentially constant in a local neighborhood of the pixel under consideration. The optical flow is then determined by solving the following system of linear equations for each pixel in a window:

4 $$\begin{aligned} A v = b, \end{aligned}$$

5 $$\begin{aligned} A = \begin{bmatrix} \frac{\partial I}{\partial x}(x_1, y_1) & \frac{\partial I}{\partial y}(x_1, y_1) \\ \frac{\partial I}{\partial x}(x_2, y_2) & \frac{\partial I}{\partial y}(x_2, y_2) \\ \vdots & \vdots \\ \frac{\partial I}{\partial x}(x_n, y_n) & \frac{\partial I}{\partial y}(x_n, y_n) \end{bmatrix}, \quad v = \begin{bmatrix} u \\ v \end{bmatrix}, \quad b = \begin{bmatrix} -\frac{\partial I}{\partial t}(x_1, y_1) \\ -\frac{\partial I}{\partial t}(x_2, y_2) \\ \vdots \\ -\frac{\partial I}{\partial t}(x_n, y_n) \end{bmatrix}. \end{aligned}$$

Here $A$ is the matrix of image gradients, $b$ represents the matrix of negative temporal gradients of the image intensity at different points and $\textbf{v} = [u \; v]^T$ is the optical flow vector. A solution can be obtained using the Moore-Penrose inverse^32,33^ $A^+$ of *A*:

6 $$\begin{aligned} \textbf{v} = A^+\textbf{b}= (A^T A)^{-1} A^T \textbf{b}. \end{aligned}$$

### Computer vision system for prospective gating

**Figure 2:**
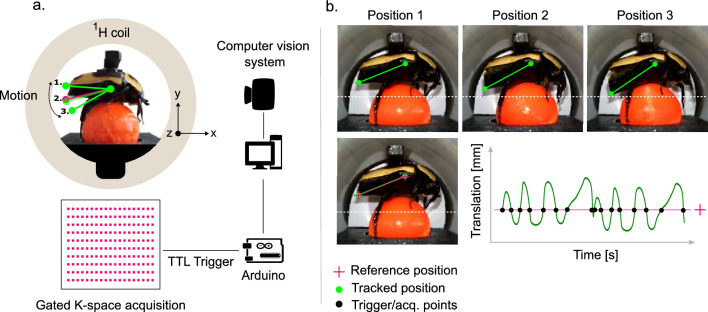
In situ computer vision system for prospective gated MRI (figure created using Inkscape v1.3.2 (https://inkscape.org)). (**a**) Schematic of computer vision based prospective gating. (**b**) Abdominal motion phases of the insect..

**Figure 3:**
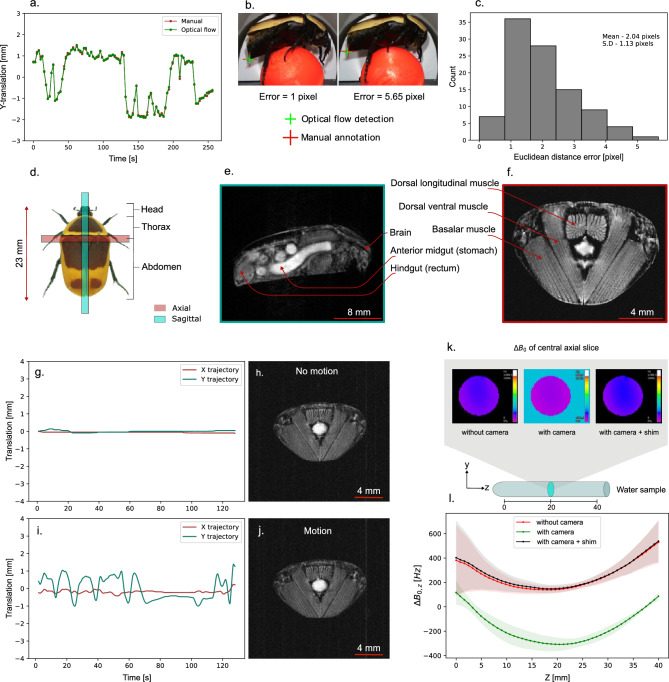
Euclidean distance error between optical flow algorithm detection and manual annotation for 100 images. (**a**) Y-position trajectory. (**b**) Sample images showing small and large error. (**c**) Error distribution. Insect anatomical MRI for reference. (**d**) MRI slice position. (**e**) Sagittal MRI showing the abdomen. (**f**) Axial MRI showing the thorax of the insect. (**g**) Motion trajectory of the inactive insect abdomen. (**h**) Thorax MRI of the inactive insect. (**i**) Motion trajectory of the walking insect abdomen. (**j**) Thorax MRI of the active insect. $\Delta B_0$ distribution with and without the camera system, and with camera + active shim for a water sample. (**k**) Central axial slice. (**l**) Coronal slice [z-axis].

A prospective gating strategy for acquiring k-space lines has been incorporated for the motion compensated imaging of walking insect on a treadmill. The integrated optical imaging system with the real time tracking using the developed computer vision algorithm provide the triggering signal for the gated k-space acquisition. We are using an OpenCV^34^ based implementation of sparse optical flow computation algorithm for the tracking of the defined body part of the insect. The posterior part of the insect abdomen as shown in Fig. 3b is the body part or keypoint tracked throughout the study, unless specified otherwise. The current motion model in consideration only requires 2D motion information. The algorithm compares the tracked position with the predefined reference position or pose of the insect and generates triggering signal via the serially connected Arduino uno microcontroller (Arduino AG, Ivrea, Italy) whenever the position is within the defined limit. This allows acquisition of the k-space lines in a spatially consistent manner, thereby reducing gross rigid body motion of the insect. The complete control strategy is provided in Algorithm 1 with the schematic diagram in Fig. 2a.

**Algorithm 1:**
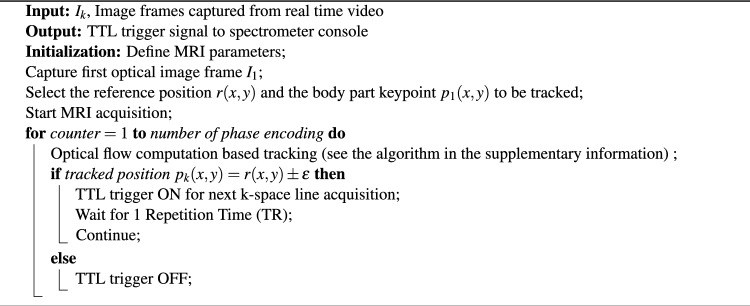
Computer vision based prospective gating

### Computer vision system benchmarking

The proposed optical imaging based prospective gating strategy does not need any cross-calibration procedures as common in the prospective motion correction literature in clinical MRI^3^. The triggering pulse generation pipeline computes the position match of the defined keypoint between image frames in the same coordinate system and do not need a coordinate transformation.

For testing the accuracy of the optical flow computation based algorithm for detection and tracking of the keypoint, we compared the algorithm detection with manually annotated ground truth data. We extracted 100 images from a 256 s long video of in situ behaving insect for the ground truth annotation. The optical flow algorithm described in “Optical flow based tracking” section tracks the keypoint defined for the 100 images. The Y-position trajectory for the algorithm detection and human annotation is shown in Fig. 3a. The error is quantified using the Euclidean distance between the manual and algorithm detection of the keypoint (see example images in Fig. 3b), with mean and standard deviation has been found out to be $2.04 \pm 1.13$ pixels (or ${91}\,\upmu \hbox {m} \pm {50}\,\upmu \hbox {m}$) for the 100 images, with the corresponding histogram shown in Fig. 3c. For accounting the human variability in annotation, we also found the Euclidean distance error between two separate trails of annotations of the same data to be $1.47 \pm 0.85$ pixels (or ${66}\,\upmu \hbox {m} \pm {38}\,\upmu \hbox {m}$). We can see that the developed optical flow based algorithm detection has close to human level accuracy in detecting the keypoint.

There is inherent latency in the computer vision based triggering system. This is due to the combined effect of limited frame rate of the camera, extension cable to the camera and the computer, optical flow computation for the tracking, serial communication with the Arduino and TTL trigger to the spectrometer for one scan of MRI k-space. The total latency has been calculated from individual segments starting from the acquisition of first video frame to the TTL trigger acquisition of first k-space line, and found to be 43 ms.

### Animal handling and ethical statement

We used a live adult sun beetle (*Pachnoda marginata* (Drury, 1773)) beetle specimen for the study, which was acquired from an online petstore (https://thepetfactory.de), kept inside a custom made terrarium at room temperature, and fed fruit and water as diet, as specified by the vendor. We used a UV-curable adhesive to tether the insect. Initially, the insect was placed inside a ventilated container and immobilized by cooling to ${2}^{\circ }\hbox {C}$ for 15 minutes. A single drop of the adhesive was applied on the 3D printed PLA-based tether, which was then positioned on top of the dorsal region of the beetle’s body approximately near the scutellum. This setup was placed on a platform under a UV lamp, which was activated for 10 seconds to cure the adhesive. Following curing, the tethered insect was gently transferred onto the spherical treadmill setup. The visual inspection confirmed the well-being of the insect following the brief UV exposure. The exposure was found out to be non-damaging, as the insect, after basking in the UV light, awoke from a state of dormancy and became active. The tethered insect on the treadmill was carefully positioned inside an RF volume coil for the MRI experiments. Once the experiments were completed, the adhesive tether was easily removed using a cotton swab soaked in warm water. The adhesive did not leave any residue on the insect body. The live insect was subsequently checked for any signs of damage and returned to the terrarium. The same insect was used for multiple subsequent trails of imaging experiments, and euthanized after the study.

## Results

### Effect of in situ optical imaging system on MRI

Integrating the optical imaging system inside the high-field magnet might introduce distortions to the $B_0$ field. This can be characterised using $B_0$ field map experiments with a uniform water phantom sample. The camera system is placed 45 mm from the center of the RF volume coil as shown in Fig. 1b. We used a sample test tube of diameter 17 mm and length of 120 mm uniformly filled with distilled water and placed inside the 35 mm $^{1}H$ volume coil. $B_0$ field map experiments were carried out with and without the live optical imaging system inside the magnet. An additional $B_0$ map experiment in the presence of the optical imaging system with active shimming has been carried out to see how far the static $B_0$ field distortion can be corrected.

We used the standard *FieldMap* sequence available with the Paravision 360 v3.3 (Bruker BioSpin GmbH, Ettlingen, Germany) software for the experiments. All the field map experiments are carried out with an image size of 128 $\times$ 128 $\times$ 64 voxels with a FOV of 40 mm $\times$ 40 mm $\times$ 40 mm and an in plane resolution of 312 ${\upmu \hbox {m}}$ $\times$ 312 $\upmu \hbox {m}$, echo time of 1.529 ms, repetition time of 35 ms with no signal averaging and total acquisition time is 276 s. The $B_0$ field distribution of the central axial slice for the 3 aforementioned cases is shown in Fig. 3k. Mean and standard deviation of $B_0$ field variation of each slice $\Delta B_{0,z}$ has been calculated and plotted to characterize field inhomogeneity along the Z-direction, as shown in Fig. 3l. The presence of the camera introduces an approximately 450 Hz lateral shift in the $B_0$ field distribution, as evident from the measured $B_0$ map in Fig. 3l. This shift appears to be static and effectively corrected using active shimming with the *Study Shim* routine in Paravision 360v3.3, as shown in Fig. 3k, l.

**Figure 4:**
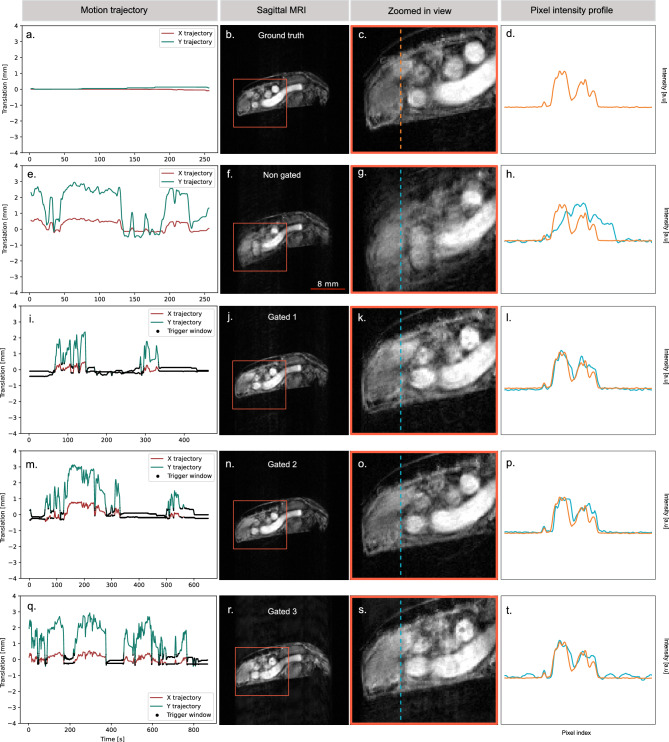
Prospective gating for motion compensated sagittal MRI of the behaving insect. (**a**) Motion trajectory of the inactive insect abdomen keypoint extracted using the in situ computer vision system. (**b**) Sagittal MRI of the inactive insect on treadmill. (**c**) Zoomed-in view showing the insect abdomen without motion artifacts. (**d**) Corresponding pixel intensity profile. (**e**) Motion trajectory of the behaving insect. (**f**) Sagittal MRI of the behaving insect. (**g**) Zoomed-in view showing the behaving insect abdomen with motion artifacts. (**h**) Corresponding pixel intensity profile. (**i**) Motion trajectory of the of the behaving insect with prospective gating (iteration 1). (**j**) Sagittal MRI of the behaving insect with prospective gating. (**k**) Zoomed-in view showing the insect abdomen with reduced motion artifacts. (**l**) Corresponding pixel intensity profile. (**m**) Motion trajectory of the of the behaving insect with prospective gating (iteration 2). (**n**) Sagittal MRI of the behaving insect with prospective gating. (**o**) Zoomed-in view showing the insect abdomen with reduced motion artifacts. (**p**) Corresponding pixel intensity profile. (**q**) Motion trajectory of the of the behaving insect with prospective gating (iteration 3). (**r**) Sagittal MRI of the behaving insect with prospective gating. (**s**) Zoomed-in view showing the insect abdomen with reduced motion artifacts. **(t)** Corresponding pixel intensity profile.

### MRI of the inactive insect

MRI of the inactive insect for the anatomical reference has been shown in main text Fig. 3e, f. Key internal anatomical structures, including the brain, digestive system components, and major thorax muscle groups are labeled for ease of comparison in the subsequent sections. We used the treadmill setup without pneumatic activation of the ball for imaging, with the insect tethered but inactive. For imaging the thorax region of the insect (Fig. 3f), we used the RARE sequence (Paravision 360 v3.3, Bruker BioSpin GmbH, Ettlingen, Germany) with a short echo time of 5.78 ms, repetition time of 3000 ms, slice thickness of 1 mm, image size of 1024 $\times$ 1024 pixels with a FOV of 20 mm $\times$ 20 mm and an in plane resolution of 20 ${\upmu \hbox {m}}$ $\times$ 20 ${\upmu \hbox {m}}$, 5 signal averages, RARE factor of 3 and total acquisition time of 1 hour 25 minutes 15 seconds for one image. For the sagittal section (Fig. 3e), we used the RARE sequence with a short echo time of 5.24 ms, repetition time of 1000 ms, slice thickness of 1 mm, image size of 256 $\times$ 256 pixels with a FOV of 30 mm $\times$ 30 mm and an in plane resolution of 117 ${\upmu }\hbox {m}$ $\times$ 117 ${\upmu }\hbox {m}$, no signal average, RARE factor of 1 and total acquisition time of 256 s for one image.

### MRI of the behaving insect

The spatial and temporal characteristics of the motion are highly significant when it comes to strategies for motion correction. The overall motion of the walking insect on a treadmill is a combination of leg motion, gross body motion due to semi periodic abdominal motion, head motion and internal organ dynamics associated with the digestive system and hemolymph circulation. As a proof-of-concept study, we are focusing on the reduction of motion artefact due to the gross abdominal motion of the active insect, which is mainly constrained to the imaging plane. Three different phases of the semi-periodic abdominal motion has been shown in the Fig. 2b for representation. We consider the inter-scan motion where the motion mostly take place in between the phase encoding steps of MRI acquisition. The echo time (TE) and echo signal sampling time is in the order of milliseconds and the motion in consideration is minimal during these period and can be neglected.

We consider two major cases for the MRI of the walking insect. One axial section of the thorax region, which has the least amount of motion and a sagittal section longitudinally through the whole body of the insect, which has considerable amount of motion artifacts due to the abdominal motion, schematic of the sections are shown in Fig. 3d. We used the RARE sequence for all the imaging experiments unless mentioned otherwise. The spin echo based imaging sequence helps in reducing the severe susceptibility artefacts due to the hard exoskeleton of the insect. For imaging the thorax region of the insect, we used a short echo time of 5.24 ms, repetition time of 1000 ms, image size of 512 $\times$ 512 pixels with a FOV of 20 mm $\times$ 20 mm and an in plane resolution of 39 ${\upmu }\hbox {m}$ $\times$ 39 ${\upmu }\hbox {m}$, slice thickness of 1 mm, no signal average, RARE factor of 4 and total acquisition time of 128 s for one image. Figure 3h shows the thorax MRI when the insect was inactive with minimal motion as evident from the motion trajectory (see Fig. 3g) of the abdomen, and Fig. 3j shows the thorax MRI of the actively walking insect, with the corresponding motion trajectory in Fig. 3i. Both images were acquired without triggering. There is no visible difference between the images and this indicates the fact that as far as the motion is out of the excited slice, it is possible to acquire motion free MRI images of the active insect with out any active motion correction strategies.

For the sagittal MRI section, we used a short echo time of 5 ms, repetition time of 1000 ms, image size of 256 $\times$ 256 pixels with a FOV of 30 mm $\times$ 30 mm and an in plane resolution of 117 ${\upmu }\hbox {m}$ $\times$ 117 ${\upmu }\hbox {m}$, slice thickness of 1 mm, with no signal average, RARE factor of one and total acquisition time of 256 s for one image. The sagittal section MRI with the corresponding motion trajectory (Fig. 4e), without gating is shown in Fig. 4f, the zoomed in view to the right in Fig. 4g clearly shows the extend of abdominal motion induced artifact. Figure 4j, n, r shows the same sagittal section MRI with corresponding zoomed in view in Fig. 4k, o, s, when the prospective gating has been employed for different motion trajectories (shown in Fig. 4i, m, q). Figure 4b shows the same sagittal section MRI of the inactive insect with zoomed in view in Fig. 4c, showing least amount of motion artifacts with corresponding motion trajectory shown in Fig. 4a, as reference image for comparison. The pixel intensity profiles of the corresponding sagittal MRI as shown in Fig. 4d, h, l, p, t demonstrate the extent of motion artifacts and the effectiveness of correction using gating. We can see evident reduction in motion artifacts in the gated MRI results. There is a variation in the acquisition time of the different prospective gated MRI compared to reference non-gated and inactive insect MRI. This variation is due to the non-uniform waiting periods between triggering instances, which are heavily influenced by the behavior of the walking insect.

Observable differences in SNR between thoracic MRI (Fig. 3h, j) and abdominal MRI (Fig. 3e) are due to both variations in acquisition parameters and anatomical characteristics. Although both scans use comparable TR and TE values, the abdominal MRI exhibits higher SNR due to its larger voxel size, single-echo acquisition (RARE factor = 1), and larger imaging volume, all of which contribute to a stronger overall signal. In contrast, the thoracic MRI prioritizes higher spatial resolution with smaller voxel size and employs a RARE factor of 4, resulting in longer echo trains and increased $T_{2}$ decay effects, which collectively reduce SNR. For the abdominal sagittal MRI, a RARE factor of 1 is used to minimize sensitivity to motion between echoes during gated acquisition. Large RARE factor means multiple echoes will be acquired per gating event, which is more sensitive to motion. For the thoracic MRI, there was no gating and hence used an RARE factor of 4, which is a standard choice to accelerate RARE based acquisitions. Gating can be applied with larger RARE factors, but this requires sequence modifications and precise timing to control increased motion sensitivity from longer echo trains. A prolonged repetition time (TR) was used in all the imaging experiments to ensure full $T_{1}$ relaxation, enhancing signal intensity and image contrast, and also to accommodate system latency between trigger signals. Mild ghosting artifacts observed in the gated abdominal MRI arise from a combination of residual motion and system latency. This is particularly evident in the gated scan labeled “Gated 3” (see Fig. 4r), which exhibits a pronounced motion profile. The triggering system has a latency of 43 ms, and any rapid physiological motion during this delay contributes to the persistence of these artifacts.

## Discussion

The in situ treadmill setup with the integrated computer vision based prospective gating technique shows clear reduction in motion artifacts, thereby allowing MR imaging of a behaving insect. The accuracy and latency of the developed computer vision system for the motion estimation is within the limits for the successful application to the current motion model, but this can be improved to a greater extent with state of the art high speed computer vision systems and faster processing algorithms. This proof-of-concept study focused on the development of treadmill-based experiments within the domain of MR microscopy. Our work specifically addressed gross abdominal motion in the insect. Extending the same approach to other body parts is feasible, provided that their motion can be accurately estimated and quantified. In our current setup, the impact of leg motion on the MRI signal appears to be negligible within the selected imaging slices. Head motion is another important aspect of insect locomotion; however, its effects are mostly localized and can be neglected in our study. The abdominal motion model is semi-periodic and the acquisition may take more time due to waiting periods for the position match for triggering, which is analogous to the respiratory triggering in clinical MRI. One way to address this challenge is to use computational frameworks that reconstruct large-scale 3D dynamic MRI from rapid, continuous, non-gated acquisitions. These approaches leverage compressed low-rank modeling and stochastic optimization to handle severe undersampling and irregular motion, with demonstrated effectiveness in pulmonary and dynamic contrast-enhanced imaging^35^. Our developed experimental setup is particularly well-suited for such approaches, as it enables the integration of accelerated k-space sampling and advanced reconstruction techniques to achieve real-time temporal resolution. This capability enables biomechanical studies of insect body pose and locomotion, similar to human studies that capture vocal tract dynamics during speech and sustained phonation^36,37^. Moreover, the current method can be extended as a retrospective gating approach where k-space is oversampled for multiple image frames of the same slice during the motion period, and the synchronization or binning of k-space lines with respect to the motion phases in post processing can effectively reduce the artefacts. This can avoid the irregular waiting periods between triggering instances and also provide multiple MRI frames enabling dynamic MRI of the behaving insect.

This study does not address internal organ motion, which can also contribute to image blurring, although typically to a lesser extent than whole-body movement. MR-based techniques such as navigators^18,19,38^ can be used to estimate internal organ motion by acquiring additional data during scanning, enabling both prospective and retrospective correction. A further challenge lies in the spatial constraints imposed by the RF coil and magnet bore during imaging of behaving animals. This has been addressed using the treadmill-based setup introduced in this study. However, such a configuration reduces the effective filling factor. Custom-designed coil geometries tailored to the specific model organism could help overcome this limitation by enhancing the filling factor, signal-to-noise ratio, and overall sensitivity.

## Outlook

While advanced techniques such as high-speed X-ray cine-tomography have successfully captured 4D internal dynamics in small, living insects^39,40^, and modalities like fluorescence imaging, optogenetics, and electrophysiology have proven effective in studying behaving vertebrate and invertebrate model organisms^41–43^, the majority of these methods remain invasive to varying degrees. This reinstates the argument for the use of magnetic resonance-based methods, due to its inherent non-invasiveness and versatility. Despite their potential, MR methods remain an underexplored field in invertebrate zoology, largely due to a lack of dedicated methodological developments. Consequently, most existing studies are confined to immobile, sedated, or deceased animals^44^. This is mainly due to the nonidealities of using MR-based techniques for studying live, behaving model invertebrates. The small size of these organisms makes MRI less sensitive at such scales, but employing higher magnetic field strengths, like the 15.2 T field used in this study, is a promising approach to enhance the signal-to-noise ratio (SNR) and spin sensitivity. However, the developed methodology is not inherently restricted to the 15.2 T system and can be adapted for other high-field platforms commonly used in preclinical MRI. Extension to lower-field systems or those with limited gradient performance is also feasible, with appropriate consideration of spatial resolution and SNR trade-offs. These limitations can be mitigated to some extent through longer signal averaging or the use of custom hardware, such as tailored RF and gradient coils.

Recent advancements in computer vision for markerless tracking of animal behavior have been profoundly impactful across various branches of zoology. State-of-the-art systems now employ sophisticated deep learning methods to analyze video recordings and identify intricate behaviors with great precision^45,46^. The present study solely focused on the motion estimation aspect for the artefact reduction. However, the MR-compatible computer vision system developed here could also be adapted for quantifying organism behavior, either by modifying the current optical flow–based algorithm or by incorporating deep learning-based methods. The chemical composition of living matter can be detected and quantified using Magnetic Resonance Spectroscopy (MRS), a complementary technique to MRI. Integrating in situ behavior estimation with localized *in vivo* MRS offers unique opportunities for studying both function and metabolism, potentially providing insights into the biochemical processes underlying organism behavior. In conclusion, traditional preclinical small-animal MRI research methodologies are developed under the assumption that motion is unavoidable, inherently imposing limitations on the breadth of research frontiers that can be explored when studying live, behaving model organisms. Our method represents an initial step in mitigating these limitations.

## Supplementary Information

Supplementary Information 1.

Supplementary Information 2.

## Data Availability

The code and datasets used in this study are publicly available at: https://github.com/chenakkara/Treadmill_MRI.git.
