# Supplementary material for: Motion compensated magnetic resonance imaging of an active sun beetle using an in situ treadmill
Source: Sci Rep. 2025 Nov 18;15:40340. doi: 10.1038/s41598-025-27800-5 (PMC12627492; doi:10.1038/s41598-025-27800-5)
Supplement: Supplementary file 1 — Supplementary Information 1. [file 41598_2025_27800_MOESM1_ESM.pdf]

Supplementary information for
"Motion compensated magnetic resonance imaging of an active sun beetle using an *in situ* treadmill"

Ajmal Chenakkara[1], Mazin Jouda[1], Ulrike Wallrabe[2], and Jan Korvink[1,*]

[1]Institute of Microstructure Technology (IMT), Karlsruhe Institute of Technology
[2]Institute of Microsystem Technology (IMTEK), University of Freiburg
[*]Email: jan.korvink@kit.edu

## 1 Sparse optical flow based tracking algorithm

**Algorithm 1:** Lucas-Kanade Based Sparse Optical Flow Tracking Algorithm

**Input:** First frame $I_1$, selected point $p = (x, y)$, window size $W = 20 \times 20$
**Output:** Tracked point position in subsequent frames

```
/* Initialization                                                    */
```
Capture the first frame $I_1$ from the live video stream;
Select a point $p = (x, y)$ in $I_1$ to track;
Define the window size $W = 20 \times 20$;
```
/* Pre-compute Gradients                                             */
```
Compute the partial derivatives $\frac{\partial I}{\partial x}$ and $\frac{\partial I}{\partial y}$ for $I_1$ using Sobel operators;
Compute the temporal partial derivative $\frac{\partial I}{\partial t}$ between $I_1$ and the next frame $I_2$;
```
/* Track the point through subsequent frames                         */
```
**for** *each subsequent frame* $I_{k+1}$ **do**

Extract the $W$ window centered at $p_k = (x_k, y_k)$ from $I_k$;
Compute the partial derivatives $\frac{\partial I}{\partial x}$, $\frac{\partial I}{\partial y}$ and temporal partial derivative $\frac{\partial I}{\partial t}$ within the window;
```
/* Formulate the optical flow equations                              */
```
$$\begin{bmatrix} \frac{\partial I}{\partial x}(p_1) & \frac{\partial I}{\partial y}(p_1) \\ \frac{\partial I}{\partial x}(p_2) & \frac{\partial I}{\partial y}(p_2) \\ \vdots & \vdots \\ \frac{\partial I}{\partial x}(p_n) & \frac{\partial I}{\partial y}(p_n) \end{bmatrix} \begin{bmatrix} u \\ v \end{bmatrix} = - \begin{bmatrix} \frac{\partial I}{\partial t}(p_1) \\ \frac{\partial I}{\partial t}(p_2) \\ \vdots \\ \frac{\partial I}{\partial t}(p_n) \end{bmatrix}$$
;
```
/* Solve using least squares                                         */
```
$$\begin{bmatrix} u \\ v \end{bmatrix} = - \left( \begin{bmatrix} \frac{\partial I}{\partial x} & \frac{\partial I}{\partial y} \end{bmatrix}^T \begin{bmatrix} \frac{\partial I}{\partial x} & \frac{\partial I}{\partial y} \end{bmatrix} \right)^{-1} \begin{bmatrix} \frac{\partial I}{\partial x} & \frac{\partial I}{\partial y} \end{bmatrix}^T \begin{bmatrix} \frac{\partial I}{\partial t} \end{bmatrix}$$
;
Update the point position $p_{k+1} = (x_k + u, y_k + v)$ for the next frame $I_{k+1}$;

**end**

## 2 Supplementary video - *In situ* optical imaging and tracking

Video showing the *in situ* behaving insect on the treadmill, with optical flow based tracking of the posterior of the abdomen. Playback speed X1.

## 3 MR-compatible camera

The commercial webcam Microsoft LifeCam HD-3000 was modified for MR-compatibility by replacing its metallic components with non-magnetic materials. All original metallic screws connecting the camera's body to its printed circuit board (PCB) were removed and replaced with a hot glue adhesive to ensure structural stability. The integrated microphone was also removed. The modified camera was then housed within a custom 3D-printed mount, as depicted in Fig. 1(d) of the main text, and secured with polymer screws. For illumination, a single surface-mount white LED (2 mm, 5 V rigid strip type) has been glued to the camera mount, and driven using the camera's power supply.
